# Preventive Vitamin D Supplementation and Risk for COVID-19 Infection: A Systematic Review and Meta-Analysis

**DOI:** 10.3390/nu16050679

**Published:** 2024-02-28

**Authors:** Marina Sartini, Filippo Del Puente, Martino Oliva, Alessio Carbone, Nicoletta Bobbio, Elisa Schinca, Luana Giribone, Maria Luisa Cristina

**Affiliations:** 1 Department of Health Sciences, University of Genoa, 16132 Genoa, Italy; martinooliva@outlook.it (M.O.); ale_carbo@live.it (A.C.); elisa.schinca@unige.it (E.S.); luanagiribone@gmail.com (L.G.); maria.luisa.cristina@galliera.it (M.L.C.); 2 Operating Unit Hospital Hygiene, Galliera Hospital, 16128 Genoa, Italy; 3 Department of Infectious Diseases, Galliera Hospital, 16128 Genoa, Italy; nicoletta.bobbio@galliera.it

**Keywords:** vitamin D, COVID-19, prevention

## Abstract

Over the past few decades, vitamin D has been found to play a crucial role in bone homeostasis, muscle function, oncogenesis, immune response and metabolism. In the context of the COVID-19 pandemic, numerous researchers have tried to determine the role vitamin D might play in the immune response to the virus. The aim of this systematic review and meta-analysis is to demonstrate that preventive vitamin D supplementation can play a protective role in the incidence of COVID-19, mortality and admission to intensive care units (ICUs). A comprehensive search on the PubMed/MEDLINE, Scopus, Cochrane and Google Scholar databases was performed on 15 May 2023, and two of the authors independently screened the literature. As effect measures, we calculated the Odds Ratios with their corresponding 95% confidence intervals (ICs). The assessment of potential bias and the evaluation of study quality will be conducted independently by two researchers. Sixteen publications were selected for inclusion in the meta-analysis. Our findings indicate that vitamin D supplementation has a protective effect against the incidence of COVID-19 in RCT studies (OR 0.403, 95% IC 0.218, 0.747), in the incidence of COVID-19 in analytical studies (OR = 0.592, 95% IC 0.476–0.736) and in ICU admission (OR 0.317, 95% IC 0.147–0.680). Subsequent analyses were conducted by type of subject treated (patient/healthcare workers) and type of supplementation (vitamin D vs. placebo/no treatment or high dose vs. low dose). Our meta-analysis suggests a definitive and significant association between the protective role of vitamin D and COVID-19 incidence and ICU admission.

## 1. Introduction

Vitamin D is a fat-soluble vitamin and is synthesized in the epidermis. In order to become active, it requires further metabolic processes. These processes occur in the liver via 25-hydroxylation and in the kidney. The primary end product of this process is 1,25-dihydroxyvitamin D, which binds to the vitamin D receptor (VDR). The majority of the effects of vitamin D are mediated by the VDR, which promotes the expression of genes containing specific DNA sequences and is expressed in almost all nucleated cells [1]. The resulting interplay between vitamin D, VDR and the promoter/repressor proteins plays a crucial role in influencing bone mineral density, with its deficiency causing rickets and osteomalacia in children and osteomalacia in adults. The interaction of vitamin D with its receptor increases the efficiency of intestinal calcium absorption from 10–15% to 30–40% and phosphorus absorption from 60 to 80%. Its deficiency causes a decrease in intestinal calcium absorption and an increased parathyroid hormone (PTH) level. PTH activates the transformation of preosteoclasts to mature osteoclasts, which dissolves the collagen matrix in bone and causes phosphaturia, thus reducing the mineralization of the collagen matrix [2].

Vitamin D sufficiency is normally estimated by measuring 25 hydroxyvitamin D (25[OH]D); however, the optimal serum vitamin D level for skeletal health is controversial, mostly because the target may vary by stage of life and ethnicity [3,4,5]. The National Institutes of Health suggest that the skeletal health of people with vitamin D concentrations below 30 nmol/L (12 ng/mL) is at risk for vitamin D insufficiency, whereas this condition is also expected for the skeletal health of some people with vitamin D concentration levels of less than 50 nmol/L (20 ng/mL) [6].

For other authors, the minimum desirable concentration for the skeletal health of vitamin D ranges between 70 and 80 nmol/L [7]. As approximately 3 percent of the human genome is under the control of 1,25-dihydroxyvitamin D, the activity of vitamin D has been theorized to be involved in the regulation of other systems, such as muscle function, oncogenesis, immune response and metabolism. However, since there is no clear evidence in vivo regarding the potential advantages of vitamin D in the regulation of these systems, the establishment of a definitive cut-off value for vitamin D levels is still under scrutiny [8].

In the context of the association between vitamin D and infection, it is important to note that a definitive correlation between the impact of diminished or elevated levels of vitamin D and the occurrence or gravity of the infection remains elusive. On the other hand, vitamin D has been observed to attenuate the activation of the acquired immune system, to contribute to the synthesis of defensins, to be pivotal for enhancing the phagocytic activity of macrophages towards pathogens [9] and to modulate the immune system response by regulating the inflammatory cascade [10].

The overall effect of vitamin D has nonetheless been under evaluation in different diseases. Currently, the main areas of study regarding the role of vitamin D in the response to infections concern, to varying degrees, the respiratory system (COPD exacerbations, tuberculosis, upper respiratory disease and COVID-19) [11,12]. Regarding COVID-19, there is growing interest in understanding the role of vitamin D in the immune response to the virus, especially given its unique role as a pathogen compared to other viral forms that cause respiratory tract infections [13,14,15]. Based on current findings, the role of vitamin D in COVID-19 is still under investigation. There exist data supporting the proposition that adequate levels of serum vitamin D may confer protection against COVID-19 infections, both in terms of incidence and mortality. Authors who assert this correlation have relied on observational studies wherein vitamin D levels appeared protective even after adjustments for variables such as age, gender and comorbidities [16]. However, it is important to highlight that previous research has failed to validate this hypothesis, partially because they discovered that vitamin D deficiency was secondary to other factors correlated with a higher susceptibility to SARS-CoV-2 infection [17]. The objective of this current meta-analysis is to examine whether the administration of vitamin D for any purpose prior to the onset of COVID-19 disease could yield a beneficial outcome in terms of prevalence, complications and mortality. This will be accomplished by assessing studies that specifically considered the adjustment of other variables.

## 2. Materials and Methods

This systematic review and meta-analysis of the literature was conducted and reported according to the Preferred Reporting Items for Systematic Reviews and Meta-analyses (PRISMA) statement [18] to ensure the current standards for systematic review reporting were met. The investigated question was formulated using the PICO methodology. The population comprised patients or healthcare workers supplemented with vitamin D before COVID-19 infection, with the primary outcome being COVID-19 incidence and secondary outcomes including ICU admission and mortality. The study protocol was registered on the PROSPERO database (registration number CRD42023469817) [19].

### 2.1. Data Sources and Search Strategy

A comprehensive search on the PubMed/MEDLINE, Scopus, Cochrane and Google Scholar databases was performed for a combination of keywords (“COVID-19” OR “SARS-CoV-2” OR “coronavirus” OR “2019-nCoV”) AND (“vitamin D” OR “cholecalciferol” OR “calcitriol”) using Medical Subject Heading (MeSH) terms as vocabulary, according to the National Center for Biotechnology Information (NCBI) nomenclature and guidelines and, where appropriate, a wild-card option. 

### 2.2. Selection of Studies

Inclusion criteria were (1) articles with relevant quantitative details and information on Vit D supplementation before a COVID-19 diagnosis and its protective role against COVID-19 infection, mortality and other clinically significant outcomes; (2) RCT, cohort, cross-sectional, case-control and quasi-experimental studies were considered. 

Exclusion criteria were (1) items not directly pertinent to the query string; (2) articles not containing sufficient information on the relationship between vitamin D supplementation before COVID-19 infection and outcome; (3) articles not meeting the PICOS criteria (P: patients and healthcare workers; I: patients or healthcare workers supplemented with Vit D before COVID-19 infection; C: patients or healthcare workers who received the standard dose, a lower dose, no therapy or a placebo; O: COVID-19 incidence, ICU admissions and mortality; S: RCT, cohort, cross-sectional, case–control and quasi-experimental studies were considered); all such articles were consequently discarded. No time filter or language filter was applied. For further details of the search strategy, see Table 1.

### 2.3. Data Extraction and Risk of Bias Assessment

Two authors independently screened the literature. Any case of disagreement was solved by discussion until consensus was reached. After the full test review, the papers included were retained for data extraction.

Data for the meta-analysis were extracted from the studies included by means of a standardized documentation form. The parameters extracted were the surname of the first author, the year and country of publication, the type of study, number of deaths, ICU admission, length of stay, endotracheal intubation, number of COVID-19 infections, age, sex, type of comparison performed in the study (i.e., vitamin D supplementation vs. no treatment; high-dose vs. low-dose vitamin D supplementation; vitamin D vs. placebo); duration of intervention; amount of vitamin D supplemented in the treated group and where available also in the control group; number of patients enrolled and their subdivision in the subgroup. 

As effect measures, we calculated the Odds Ratios with their 95% confidence intervals (CIs). The assessment of potential bias and the evaluation of study quality were conducted independently by four researchers employing distinct assessment tools tailored to the specific study design presented in the paper at hand. Specifically, for case series, we employed the “National Institutes of Health (NIH) quality assessment tool for case series studies”. For papers presenting cohort or cross-sectional studies, we utilized the “National Institutes of Health (NIH) quality assessment tool for observational cohort and cross-sectional studies”. In the case of case–control studies, we employed the “National Institutes of Health (NIH) quality assessment tool for case–control studies” and for the RCT, the “The National Institutes of Health (NIH) quality assessment tool of controlled intervention study” [20]. Any disagreement was solved by consensus. 

Ten studies were classified as “good” [21,22,23,24,25,26,27,28,29], six as “fair” [30,31,32,33,34,35] and one as “poor” [36].

### 2.4. Statistical Analysis

Data synthesis, both qualitative and quantitative, was undertaken by a pair of researchers. Any inconsistencies or discrepancies encountered during this process were diligently resolved through direct confrontation and contributions from all the researchers. For the meta-analysis, we employed STATA SE 18, a robust statistical software package. During the meta-analysis, we also rigorously assessed statistical heterogeneity using both the *I*^2^ statistics and the heterogeneity χ^2^ test. Heterogeneity was deemed statistically significant when the *p*-value (χ^2^) was <0.1. More specifically, we established that *I*^2^ values of 25%, 50% and 75% corresponded to low, moderate and high levels of heterogeneity, respectively. In instances where heterogeneity reached a significant level, classified as either moderate or high, we employed a random-effects model for the meta-analysis. In the case of low heterogeneity, a fixed model was used. The results of our study analysis will be presented using summary outcome and effect measures.

The ORs of the meta-analyses were deemed significant when the confidence intervals did not contain the value “1”. A narrower confidence interval than that of the individual studies indicates less imprecision. 

To identify sources of variation, further stratification was performed with respect to study quality. In addition, in the sensitivity analyses, the stability of the pooled estimate with respect to each study was investigated by excluding individual studies from the analysis. Possible publication bias was visually inspected by means of a funnel plot. If asymmetry was detected by visual assessment, exploratory analyses using trim and/or fill analysis were performed in order to investigate and adjust this. In addition, the probability of publication bias was tested by means of Egger’s linear regression, with a value of *p* < 0.05 being indicative of publication bias.

## 3. Results

**Figure 1:**
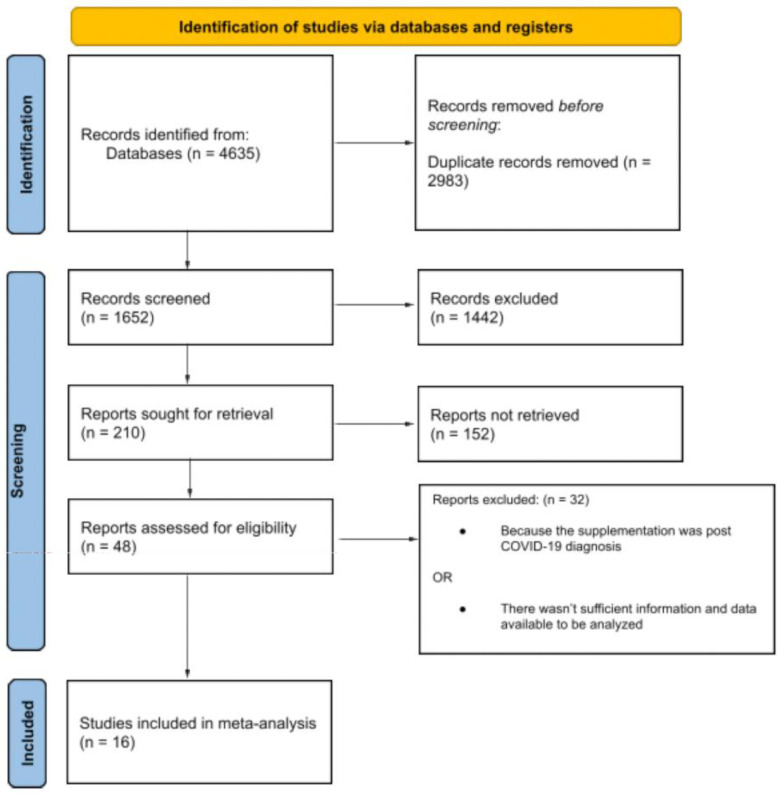
PRISMA 2020 flow diagram of study selection, inclusion and synthesis [18].

A total of sixteen publications were included in the analysis (Figure 1). Among these publications, three [24,26,29] featured two studies each. In total, seven RCTs were evaluated, while the remaining studies were categorized as analytical studies. Among the seven RCTs [21,23,25,28,29,35,36], five were conducted on HCWs [28,29,35,36], while the other two focused on patients. Among the five RCTs conducted on HCWs, four compared a population undergoing vitamin D treatment with a population not undergoing treatment [28,29,36], while one compared a high-dose vitamin D regimen with a low-dose regimen [35]. As regards the two RCTs performed on patients, one compared a treated population with an untreated one [23], and the second compared a high-dose regimen with a low-dose regimen [25]. All studies conducted on HCWs were performed as RCTs. The remaining eight analytical studies evaluated the incidence of vitamin D supplementation in a patient population. Three analytical studies assessed the ICU admission rate [22,27,31] and eleven studies evaluated mortality, with ten being analytical and one being an RCT [21,22,24,26,27,30,31,32,34].

The main characteristics of studies included in the meta-analysis are presented in Table 2. In addition to the previously discussed features, such as study design, type of participants (patients or healthcare workers) and different treatment types, Table 2 provides information on the setting of each study, along with the number of participants, age (mean ± standard deviation or median and interquartile range (IQR)) and sex (in absolute number and percentage). These details are further subdivided into the group that received vitamin D supplementation and the control group.

Finally, the outcomes (COVID-19 incidence, mortality and ICU admission) for each included study are reported. Globally, as indicated in Table 2, COVID-19 incidence was assessed in thirteen studies, mortality in eleven studies and intensive care unit admission in only three studies.

Table 3 provides details on the amounts of vitamin D administered and the type of comparison used in the two treatment arms (intervention and control) for each study, where available. The precise amount of vitamin D administered was available for 15 out of 19 studies and was reported in IUs (International Units), mg (e.g., 1 IU is equal to 0.000025 μg) or μg (e.g., 1 IU is equal to 0.025 μg). The frequency of vitamin D administration varied, with some studies using daily, weekly or monthly dosing. In 4 out of 19 studies, the amount of vitamin D administered was not reported, and in two studies, the vitamin D molecule was not mentioned.

The doses of vitamin D (cholecalciferol) administered as IU/daily were 5000, 4000, 3200, <1000 and 400; those administered as IU/weekly were 50,000 and 5600; and those administered as IU/monthly were 100,000, 90,000, 80,000, 52,000, 50,000, 25,000 and 10,000. The doses of vitamin D administered as calcifediol were 54,000 IU/monthly, 0.266 mg/monthly and 250 μg per dose. Ergocalciferol (vitamin D2) supplementation was only reported in one study. Calcitriol supplementation was also reported in only one study.

In 14 out of 19 studies, the control group did not receive vitamin D supplementation. In 2 out of 19 studies, the control group received a lower dose of vitamin D (2000 and 800 IU/d), and in 3 out of 19 studies, the control group received a placebo.

Additionally, Table 3 presents the number of events as absolute numbers (*n*/N) and percentages (%) for each analyzed outcome in both the intervention and control groups.

### 3.1. Evidence from RCTs on COVID-19 Infection Risk

**Figure 2:**
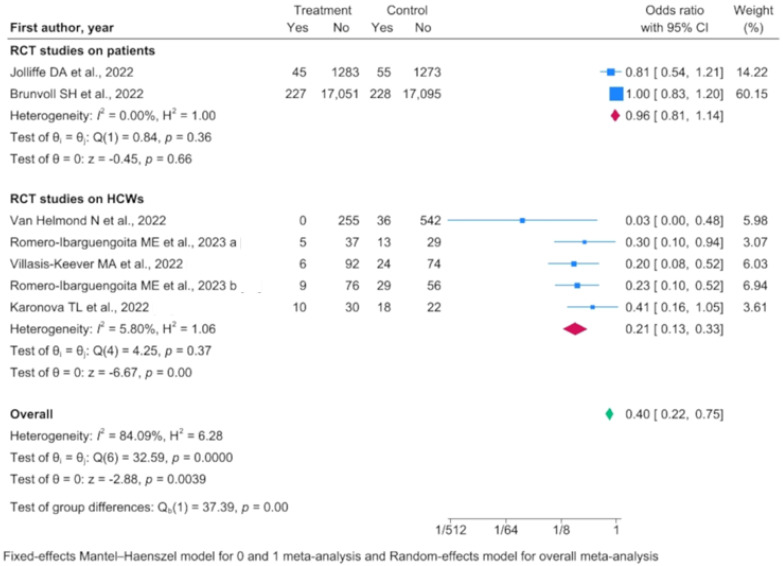
Forest plot of randomized controlled trials (RCTs) conducted separately on healthcare workers (HCWs) and patients, as well as collectively [23,25,28,29,35,36].

In the seven RCTs considered and assessed via a random-effects model, vitamin D supplementation was associated with a decreased infection risk (OR 0.403, 95% IC 0.218–0.747) despite substantial heterogeneity among the studies. Among the seven RCTs, five were conducted on healthcare workers (HCWs) and were more reliable in terms of heterogeneity (Figure 2).

In the RCTs performed on HCWs, the overall reduction in risk in the population supplemented with vitamin D was approximately 80% (OR 0.210, 95% IC 0.132–0.332). The level of heterogeneity among the studies was negligible (*I*^2^ = 5.80), and therefore a Mantel–Haenszel fixed-effects model was used. In four of the five RCTs, the follow-up time frame was adequate (at least 6 months); the only one with a shorter follow-up (45 days) was the only multicenter trial performed on HCWs. Vitamin D deficiency and insufficiency prevalence was consistent among the studies that included its measurement in their design (55–67% prevalence of vitamin D deficiency, 27–30% prevalence of vitamin D insufficiency and 6–15% of subjects presenting normal values).

Four out of the five RCTs performed on HCWs compared vitamin D supplementation with no treatment or dietary measures (treatment vs. no treatment). One evaluated the effect of a higher dosage versus a low vitamin D dosage. The treatment vs. no treatment analysis confirmed low heterogeneity among the studies and a higher protective effect of vitamin D supplementation (OR 0.177, 95% IC 0.104–0.301).

Regarding the effect of vitamin D supplementation on non-HCWs and registered by RCTs, there was no effect on the COVID-19 infection rate (OR 0.963, 95% IC 0.814–1.139). This may lead to some uncertainty concerning the effectiveness of vitamin D supplementation in the general population. However, it should be highlighted that in the study by Brunvoll, the treatment group was exposed to lower dosages of vitamin D supplementation (400 IU) in comparison to other studies. The combined low prevalence of vitamin D deficiency in the study population and low dosage of supplementation may have resulted in an absence of the effect of vitamin D supplementation.

**Figure 3:**
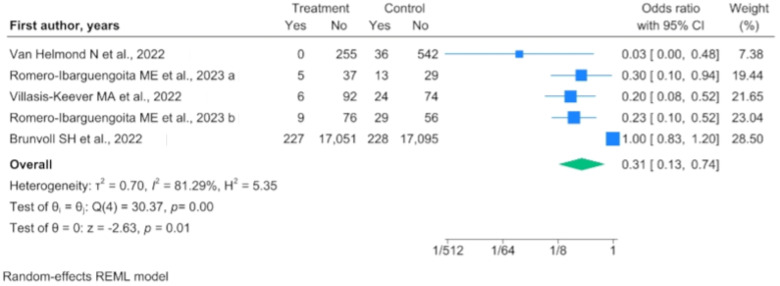
Forest plot of randomized controlled trials (RCTs) comparing vitamin D supplementation to no supplementation [23,28,29,36].

Through the evaluation of the RCT results, we observed that vitamin D supplementation resulted in a benefit for the population when compared with a population not subjected to supplementation (OR 0.307, 95% IC 0.127–0.739) (Figure 3), while this benefit was not maintained when compared with a population subjected to vitamin D supplementation on a reduced dosage (OR 0.730, 95% IC 0.505–1.055).

### 3.2. Evidence from Analytic Studies on COVID-19 Infection Risk

Since these studies analyzed people who were administered different dosages of vitamin D supplementation versus people who were not receiving treatment, there was no standardization of the intervention. Consequently, we found limited data regarding the prevalence of vitamin D deficiency and insufficiency. Conversely, the analytical studies were based on a greater number of patients and with longer study durations. The resulting meta-analysis confirmed the protective role of vitamin D supplementation (OR 0.592, 95% IC 0.476–0.736) despite high heterogeneity among the studies (*I*^2^ = 98.99).

### 3.3. Evidence from RCTs and Analytical Studies on SARS-CoV-2-Related Mortality

The only RCT evaluating vitamin D supplementation prior to COVID-19 infection and subsequent mortality was performed on 66 participants. People receiving vitamin D (in this case, a single bolus of 80,000 IU) had significantly lower mortality after adjustment for all potential confounders. No other covariables were associated with mortality (OR 0.163, 95% IC 0.0.32–0.832).

Regarding the analytical studies, no association was found between vitamin D supplementations prior to COVID-19 infection and relative mortality (OR 0.882, 95% IC 0.667–1.165). Out of the ten studies included, only three demonstrated a protective action of vitamin D against mortality in multivariable analysis. It should be noted, however, that some selection biases might limit the generalizability of the conclusions drawn from such analytical studies. In several studies, we found significant differences in the populations at baseline, despite overall homogeneity in terms of confounding variables between cases and controls. Most studies were carried out without adequate matching between cases and controls. In particular, some differences regarding BMI and age were found in two studies [27,30], while differences between the prevalence of chronic renal insufficiency, cardiovascular disease and the Charlson comorbidity index were found in another one [31]. Just three studies performed propensity score matching between cases and controls [24,26,34]. Furthermore, a high level of heterogeneity was found in the meta-analysis of the analytical studies (*I*^2^ = 93.93)

### 3.4. Evidence from Analytical Studies on ICU Admissions

**Figure 4:**
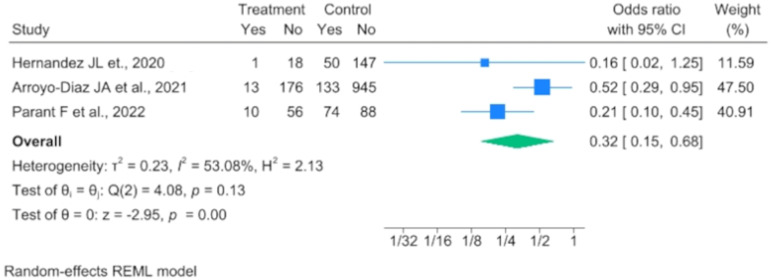
Forest plot demonstrating ability of vitamin D supplementation to reduce ICU admission [22,27,31].

Three analytical studies evaluated the effect of previous vitamin D supplementation and ICU admission due to complications of COVID-19 infection. The meta-analysis performed with the random-effects model revealed that prior vitamin D supplementation may have a protective role against ICU admission (OR 0.317, 95% IC 0.147–0.680) (Figure 4).

It should be noted that among the three studies included, one study was based on two populations with differences at baseline, while the other two were conducted on populations with a high prevalence of vitamin D deficiency/insufficiency at baseline.

## 4. Discussion

In this meta-analysis of 19 studies, consisting of 7 RCTs and 12 analytical studies encompassing a total of 1,262,235 participants, we aimed to assess the potential protective role of vitamin D supplementation before COVID-19 infection in terms of the reduction in the incidence of COVID-19 infection, ICU admission and mortality. It was possible to evaluate the incidence of COVID-19 infection in 13 out of 19 studies, mortality in 11 out of 19 studies and ICU admission in only 3 out of 19 studies.

Both RCTs and analytical studies observed a decrease in the incidence of COVID-19 infection in the population subjected to vitamin D supplementation. However, certain aspects remain to be addressed. Concerning the RCTs, limitations such as sample size and their monocentric nature may impact the generalizability of their conclusion, while the analytical studies showed a lack of data regarding the prevalence of vitamin D deficiency/insufficiency at baseline. Nonetheless, the data appear to demonstrate a protective effect of vitamin D, especially in populations with a high incidence of vitamin D deficiency/insufficiency and if HCWs are involved in the study design, presumably demonstrating higher adherence to vitamin D supplementation during the pandemic. An interesting finding that emerges from this study concerns, in addition to the high incidence of vitamin D deficiency/insufficiency in HCWs, the protective role of vitamin D even in middle-aged people. Even though this result is in line with the general consensus of literature data [37,38,39,40], it should be underlined that the evidence found is not univocal and that there remain some studies that have not confirmed the protective role of vitamin D [17,41,42]. In particular, in the study by Brunvoll, the overall effect of vitamin D was negligible. This may have been due to the low dosage of vitamin D supplemented in the treatment group and the low prevalence of vitamin D deficiency in the study group.

In terms of the severity of COVID-19, it has been observed that individuals receiving vitamin D supplementation exhibit a lower incidence of serious complications and a reduced requirement for intensive care, while no difference was noted in terms of overall mortality. It is therefore conceivable, though not yet demonstrable, that vitamin D supplementation may play a role in reducing the occurrence and complications of the pathology. However, it should be noted that the initial benefit may not be substantiated in individuals who still develop the pathology. Nonetheless, this conclusion is drawn from a small number of studies, and for this reason we believe it is useful to carry out further studies and measurements before being able to make concrete hypotheses. On the other hand, this conclusion is in line with the majority of literature data [16,43,44], despite many authors expressing concerns about the lack of robust data from large RCT series [41,42,45,46]. Additionally, it is important to highlight that much of the evidence available in the scientific literature stems from patients who received vitamin D after being diagnosed with COVID-19, which only partially overlaps with the population receiving supplementation prior to the diagnosis of the disease.

From this standpoint, Pal et al., in 2021 [47], found no association between the use of vitamin D before the COVID-19 diagnosis with intensive care unit admission and/or mortality (OR 0.71, 95% IC 0.16–3.03) in a subgroup analysis of only three studies.

Similarly, Beran et al. in 2021 [48] found, in a subgroup analysis, that there was no association between preventive vitamin D supplementation prior to COVID-19 infections and related mortality (OR 0.83, 95% IC 0.39–1.76). Although their analysis was conducted on only five studies, they found the same conclusions as our study regarding mortality. From this standpoint, the role of vitamin D supplementation and its impact on mortality in the context of different pathologies have been extensively studied. However, the conclusions of these studies are not unequivocal given the presence of issues related to the disease-attributable mortality and the role of comorbidities and confounding variables in population studies [49,50,51]. The potential protective function of vitamin D supplementation in mitigating complications associated with COVID-19 would be of heightened interest due to the potential decrease in hospitalization requirements within intensive care units. This reduction in intensive care demands could result in a consequential decrease in expenses, antibiotic therapies and the emergence of antibiotic-resistant strains, which was commonly observed during the pandemic and documented by numerous authors [52,53].

### Limitation

The main limitations of this meta-analysis concern the number of studies analyzed, which, although being greater than the number of other meta-analyses on this topic, is still not ample. However, it should be noted that the previous meta-analyses were also partly focused on different aspects, such as the administration of vitamin D during COVID-19 infection and not before, like in our study. We should also point out that there is little evidence from studies regarding ICU admission. The remaining limitations correspond to those present in the studies analyzed (first of all, the absence of sample size calculation in several RCTs and, secondly, the retrospective nature of several analytical studies). Further limitations of the studies are the absence, in several studies, of data on the prevalence of vitamin D deficiency/insufficiency in the population at baseline and, finally, the use of various formulations of vitamin D (cholecalciferol and/or calcitriol) at different dosages, which further complicate the evaluation of the effect of vitamin D supplementation, as this is carried out at different dosages in populations with different rates of vitamin D deficiency/insufficiency.

## 5. Conclusions

To the best of our knowledge, a strength of our study lies in the fact that it is the first meta-analysis conducted exclusively on studies that considered preventive vitamin D supplementation in people not affected by COVID-19 infection. Prior to our investigation, as reported in the Discussion, only two meta-analyses, which mainly focused on vitamin D supplementation post COVID-19 diagnosis, included subgroup analyses conducted on vitamin D supplementation pre COVID-19 diagnosis. We chose not to include studies in which vitamin D supplementation occurred following the diagnosis of COVID-19 infection in order to evaluate the actual impact on the incidence of the disease.

Finally, the results of our meta-analysis seem to support the use of vitamin D, especially in populations with vitamin D deficiencies, in the prevention of COVID-19 infection and in the prevention of related complications.

## Figures and Tables

**Table 1:** Search strategy adopted in the present systematic review and meta-analysis.

Search Strategy	Details	
Search string	(“COVID-19” OR “SARS-CoV-2” OR “coronavirus” OR “2019-nCoV”) AND (“vitamin D” OR “cholecalciferol” OR “calcitriol”)	
Inclusion criteria	P (patients/population):	Patients and healthcare workers
	I (intervention/exposure):	Patients or healthcare workers supplemented with Vit D before COVID-19 infection
	C (comparisons/comparators):	Patients or healthcare workers who received the standard dose, a lower dose, no therapy or a placebo
	O (outcome):	COVID-19 incidence, ICU admissions and mortality
	S (study design):	RCT, cohort, cross-sectional, case–control and quasi-experimental studies were considered
Databases	PubMed/MEDLINE, Scopus, Cochrane and Google Scholar	
Exclusion criteria	Experimental studies investigating in vitro or animal models Study design: editorial, commentaries, expert opinions, letters to the editor, review articles, original non-prospective studies and articles with insufficient details	
Time filter	None (from inception)	
Language filter	None (any language)	

**Table 2:** Characteristics of studies included in meta-analysis.

References	Study Design, Setting	Participants	Vitamin D Supplementation Group			Control Group			Outcomes (Relevant for This Meta-Analysis)
			No.	Age	Sex, Male	No.	Age	Sex, Male	
Annweiler, G. et al., 2020 [21]	Quasi-experimental with retrospective collection of data, France	Patients	29	88 (87–93)	9 (31.0)	32	88 (84–92)	19 (59.4)	Mortality
Hernandez, J.L. et. al., 2020 [22]	Case–Control, Spain	Patients	19	60.0 (59.0–75.0)	7 (36.8)	197	61.0 (56.0–66.0)	123 (62.4)	Mortality, ICU admission
Arroyo-Diaz, J.A. et al., 2021 [31]	Cross-Sectional, Spain	Patients	189	73.3 ± 13.7	62 (32.8)	1078	63.2 ± 16.3	634 (58.8)	Mortality, ICU admission
Cangiano, B. et al., 2021 [30]	Prospective Cohort, Italy	Patients	20	NA	NA	78	NA	NA	Mortality
Cereda, E. et al., 2021 [32]	Retrospective Cohort, Italy	Patients	18	68.8 ± 10.6	16 (42.1)	152	70.5 ± 13.1	141 (49.3)	Mortality
Ma, H. et al., 2021 [33]	Prospective Cohort, USA	Patients	363	59.1 ± 8.1	141 (38.8)	7934	57.4 ± 8.6	3964 (50.0)	SARS-CoV-2 Incidence
Oristrell, J. et al., 2021 [34]	Case–Control, Spain	Patients	6252	70.2 ± 15.6	2656 (42.5)	12,504	70.7 ± 14.7	5319 (42.5)	Mortality, COVID-19 incidence
Brunvoll, S.H. et al., 2022 [23]	RCT, Norway	Patients	17,278	45.0 ± 13.5	6117 (35.4)	17,323	44.9 ± 13.4	6137 (35.4)	SARS-CoV-2 Incidence
Gibbons, J.B. et al., 2022 [24]	Retrospective Cohort, USA	Patients	33,216	58	29,130 (87.7)	33,216	58	29,097 (87.6)	Mortality, COVID-19 Incidence
	Retrospective Cohort, USA	Patients	199,498	63	179,349 (89.9)	199,498	64	179,748 (90.1)	Mortality, COVID-19 Incidence
Jolliffe, D.A. et al., 2022 [25]	RCT, UK	Patients	1346	60.7 (50.2–68.5)	506 (37.6)	1328	59.8 (50.3–67.4)	498 (37.5)	Mortality, COVID-19 Incidence
Karonova, T.L. et al., 2022 [35]	RCT, Russia	Healthcare Workers	38	34 ± 2	6 (15.8)	40	36 ± 2	6 (15.0)	COVID-19 Incidence
Oristrell, J. et al., 2022 [26]	Retrospective Cohort, Spain	Patients	108,343	70.0 ± 14.0	17,926 (16.5)	216,686	70.0 ± 14.6	35,272 (16.3)	Mortality, COVID-19 Incidence
	Retrospective Cohort, Spain	Patients	134,703	68.8 ± 14.9	29,474 (21.9)	269,406	68.8 ± 15.1	59,582 (22.1)	Mortality, COVID-19 Incidence
Parant, F. et al., 2022 [27]	Retrospective Cohort, France	Patients	66	NA	27 (40.9)	162	NA	102 (63.0)	Mortality, ICU admission
Van Helmond, N. et al., 2022 [36]	RCT, USA	Healthcare Workers	255	47 ± 12	55 (21.6)	578	50 ± 13	131 (22.7)	COVID-19 Incidence
Villasis-Keever, M.A. et al., 2022 [28]	Double-Blind RCT, Mexico	Healthcare Workers	94	36.0 (30–43)	NA	98	39.0 (31–48)	NA	COVID-19 Incidence
Romero-Ibarguengoita, M.E. et al., 2023 [29]	Prospective Quasi-Experimental, Mexico	Healthcare Workers	43	NA	17 (39.5)	42	NA	23 (54.8)	COVID-19 Incidence
	Prospective Quasi-Experimental, Mexico	Healthcare Workers	28	NA	8 (28.6)	85	NA	20 (23.5)	COVID-19 Incidence

NA = not applicable.

**Table 3:** Characteristics of the study outcomes included in the meta-analysis.

References	Treatment Arms	COVID-19 Incidence (*n*/N, %)		All-Cause Mortality (*n*/N, %)		ICU Admission (*n*/N, %)	
		Intervention	Control	Intervention	Control	Intervention	Control
Annweiler, G. et al., 2020 [21]	Intervention: 50,000 IU/month, 80,000 IU or 10,000 IU every 2–3 months (cholecalciferol); control: no vitamin D supplementation	NA	NA	2/29 10.53	10/32 31.25	NA	NA
Hernandez, J.L. et al., 2020 [22]	Intervention: (11 patients were taking cholecalciferol, 25,000 IU/monthly in 10 cases and 5600 IU/weekly in 1, and 8 patients were on calcifediol, 0.266 mg/monthly)	NA	NA	2/19 10.53	20/197 5.08	1/19 5.26	50/197 25.38
Arroyo-Diaz, J.A. et al., 2021 [31]	Intervention: regularly supplemented with vitamin D (not specified); control: no vitamin D supplementation	NA	NA	50/189 26.46	167/1078 15.49	13/189 6.88	133/1078 12.34
Cangiano, B. et al., 2021 [30]	Intervention: 25,000 IU of cholecalciferol 2 times a month; control: no vitamin D supplementation	NA	NA	3/20 15	39/78 50	NA	NA
Cereda, E. et al., 2021 [32]	Intervention: 54,000 IU/month of calciferol; control: no vitamin D supplementation	NA	NA	7/18 38.89	40/152 26.32	NA	NA
Ma, H. et al., 2021 [33]	Intervention: regularly supplemented with vitamin D (not specified); control: no vitamin D supplementation	49/363 13.50	1329/7934 16.75	NA	NA	NA	NA
Oristrell, J. et al., 2021 [34]	Intervention: regularly supplemented with vitamin D (mean daily calcitriol dose: ≤0.1 μg/d; >0.1–0.2 μg/d; >0.2–<0.4 μg/d; ≥0.4 μg/d); control: no vitamin D supplementation	328/6252 5.25	703/12,504 5.62	76/6252 1.22	208/12,504 1.66	NA	NA
Brunvoll, S.H. et al., 2022 [23]	Intervention: 5 mL/day of cod liver oil (equal to 10 μg/d or 400 IU/d of vitamin D3); control: placebo	227/17,278 1.31	228/17,323 1.32	NA	NA	NA	NA
Gibbons, J.B. et al., 2022 [24]	Intervention: regularly supplemented with vitamin D (vitamin D2 or ergocalciferol); control: no vitamin D supplementation	716/33,216 2.16	987/33,216 2.98	65/33,216 0.19	86/33,216 0.26	NA	NA
	Intervention: regularly supplemented with vitamin D (vitamin D3 or cholecalciferol); control: no vitamin D supplementation	5315/199,498 2.66	6591/199,498 3.30	462/199,498 0.23	689/199,498 0.35	NA	NA
Jolliffe, D.A. et al., 2022 [25]	Intervention: 3200 IU/day of vitamin D3; control: 800 IU/day	45/1346 3.34	55/1328 4.14	NA	NA	NA	NA
Karonova, T.L. et al., 2022 [35]	Intervention: 50,000 IU/week of cholecalciferol for 2 consecutive weeks, followed by 5000 IU/day for the rest of the study; control: 2000 IU/day	10/38 26.31	18/40 45.00	NA	NA	NA	NA
Oristrell, J. et al., 2022 [26]	Intervention: >250 μg of cholecalciferol per dose (equal to 10,000 IU); control: no vitamin D supplementation	4352/108,343 4.02	9142/216,686 4.22	716/108,343 0.66	1492/216,686 0.69	NA	NA
	Intervention: >250 μg of calcifediol per dose (equal to 10,000 IU); control: no vitamin D supplementation	5662/134,703 4.20	11,401/269,406 4.23	934/134,703 0.69	1859/269,406 0.69	NA	NA
Parant, F. et al., 2022 [27]	Intervention: <1000 IU/d or 80,000 IU or 100,000 IU every 2–3 months of cholecalciferol; control: no vitamin D supplementation	NA	NA	7/66 10.61	28/162 17.28	10/66 15.15	74/162 45.68
Van Helmond, N. et al., 2022 [36]	Intervention: 5000 IU/d of vitamin D3; control: placebo	0/255 0.00	36/578 6.23	NA	NA	NA	NA
Villasis-Keever, M.A. et al., 2022 [28]	Intervention: 4000 IU/d of cholecalciferol; control: placebo	6/94 6.38	24/98 24.49	NA	NA	NA	NA
Romero-Ibarguengoita, M.E. et al., 2023 [29]	Intervention: 52,000 IU/month of vitamin D3; control: no vitamin D supplementation	5/43 11.63	13/42 30.95	NA	NA	NA	NA
	Intervention: 90,000 IU/month of vitamin D3; control: no vitamin D supplementation	9/28 32.14	29/85 34.12	NA	NA	NA	NA

NA = not applicable.

## Data Availability

The data presented in this study are available on request from the corresponding author. The data are not publicly available due to internal regulations.

## Abbreviations

ICU	intensive care unit
IC	confidence interval
OR	Odds Ratio
PTH	parathyroid hormone
COPD	Chronic Obstructive Pulmonary Disease
HCW	healthcare worker
RCT	randomized control trial

## References

[1] Bikle D.D. (2020). Vitamin D: Newer Concepts of Its Metabolism and Function at the Basic and Clinical Level. J. Endocr. Soc..

[2] Holick M.F. (2007). Vitamin D Deficiency. N. Engl. J. Med..

[3] Institute of Medicine (IOM) (2011). Dietary Reference Intakes for Calcium and Vitamin D.

[4] Jones G., Ross A.C., Caballero B., Cousins R.J., Tucker K.L., Ziegler T.R. (2014). Vitamin D. Modern Nutrition in Health and Disease.

[5] Brown L.L., Cohen B., Tabor D., Zappalà G., Maruvada P., Coates P.M. (2018). The vitamin D paradox in Black Americans: A systems-based approach to investigating clinical practice, research, and public health—Expert panel meeting report. BMC Proc..

[6] Vitamin D Fact Sheet for Health Professionals. https://ods.od.nih.gov/factsheets/VitaminD-HealthProfessional/#:~:text=Serum%20concentrations%20of%2025(OH)D%20and%20health&text=Some%20people%20are%20potentially%20at,are%20sufficient%20for%20most%20people.

[7] Dawson-Hughes B., Heaney R.P., Holick M.F., Lips P., Meunier P.J., Vieth R. (2005). Estimates of optimal vitamin D status. Osteoporos. Int..

[8] Bouillon R., Marcocci C., Carmeliet G., Bikle D., White J.H., Dawson-Hughes B., Lips P., Munns C.F., Lazaretti-Castro M., Giustina A. (2018). Skeletal and Extraskeletal Actions of Vitamin D: Current Evidence and Outstanding Questions. Endocr. Rev..

[9] Bouillon R., Quesada-Gomez J.M. (2021). Vitamin D Endocrine System and COVID-19. JBMR Plus.

[10] Gunville C.F., Mourani P.M., Ginde A.A. (2013). The Role of Vitamin D in Prevention and Treatment of Infection. Inflamm. Allergy-Drug Targets.

[11] Nnoaham K.E., Clarke A. (2008). Low serum vitamin D levels and tuberculosis: A systematic review and meta-analysis. Leuk. Res..

[12] Jolliffe D.A., Greenberg L., Hooper R.L., Mathyssen C., Rafiq R., De Jongh R.T., Camargo C.A., Griffiths C.J., Janssens W., Martineau A.R. (2019). Vitamin D to prevent exacerbations of COPD: Systematic review and meta-analysis of individual participant data from randomised controlled trials. Thorax.

[13] Lamers M.M., Haagmans B.L. (2022). SARS-CoV-2 pathogenesis. Nat. Rev. Microbiol..

[14] Gu J., Korteweg C. (2007). Pathology and Pathogenesis of Severe Acute Respiratory Syndrome. Am. J. Pathol..

[15] Sartini M., Del Puente F., Oliva M., Carbone A., Blasi Vacca E., Parisini A., Boni S., Bobbio N., Feasi M., Battistella A. (2021). Riding the COVID Waves: Clinical Trends, Outcomes, and Remaining Pitfalls of the SARS-CoV-2 Pandemic: An Analysis of Two High-Incidence Periods at a Hospital in Northern Italy. J. Clin. Med..

[16] Ali N. (2020). Role of vitamin D in preventing of COVID-19 infection, progression and severity. J. Infect. Public Health.

[17] Hastie C.E., Mackay D.F., Ho F., Celis-Morales C.A., Katikireddi S.V., Niedzwiedz C.L., Jani B.D., Welsh P., Mair F.S., Gray S.R. (2020). Vitamin D concentrations and COVID-19 infection in UK Biobank. Diabetes Metab. Syndr. Clin. Res. Rev..

[18] Page M.J., McKenzie J.E., Bossuyt P.M., Boutron I., Hoffmann T.C., Mulrow C.D., Shamseer L., Tetzlaff J.M., Akl E.A., Brennan S.E. (2021). The PRISMA 2020 statement: An updated guideline for reporting systematic reviews. BMJ.

[19] CRD42023469817. https://www.crd.york.ac.uk/prospero/display_record.php?RecordID=469817.

[20] Ma L.-L., Wang Y.-Y., Yang Z.-H., Huang D., Weng H., Zeng X.-T. (2020). Methodological quality (risk of bias) assessment tools for primary and secondary medical studies: What are they and which is better?. Mil. Med. Res..

[21] Annweiler G., Corvaisier M., Gautier J., Dubée V., Legrand E., Sacco G., Annweiler C. (2020). Vitamin D Supplementation Associated to Better Survival in Hospitalized Frail Elderly COVID-19 Patients: The GERIA-COVID Quasi-Experimental Study. Nutrients.

[22] Hernández J.L., Nan D., Fernandez-Ayala M., García-Unzueta M., Hernández-Hernández M.A., López-Hoyos M., Muñoz-Cacho P., Olmos J.M., Gutiérrez-Cuadra M., Ruiz-Cubillán J.J. (2021). Vitamin D Status in Hospitalized Patients with SARS-CoV-2 Infection. J. Clin. Endocrinol. Metab..

[23] Brunvoll S.H., Nygaard A.B., Ellingjord-Dale M., Holland P., Istre M.S., Kalleberg K.T., Søraas C.L., Holven K.B., Ulven S.M., Hjartåker A. (2022). Prevention of COVID-19 and other acute respiratory infections with cod liver oil supplementation, a low dose vitamin D supplement: Quadruple blinded, randomised placebo controlled trial. BMJ.

[24] Gibbons J.B., Norton E.C., McCullough J.S., Meltzer D.O., Lavigne J., Fiedler V.C., Gibbons R.D. (2022). Association between vitamin D supplementation and COVID-19 infection and mortality. Sci. Rep..

[25] Jolliffe D.A., Holt H., Greenig M., Talaei M., Perdek N., Pfeffer P., Vivaldi G., Maltby S., Symons J., Barlow N.L. (2022). Effect of a test-and-treat approach to vitamin D supplementation on risk of all cause acute respiratory tract infection and COVID-19: Phase 3 randomised controlled trial (CORONAVIT). BMJ.

[26] Oristrell J., Oliva J.C., Casado E., Subirana I., Domínguez D., Toloba A., Balado A., Grau M. (2022). Vitamin D supplementation and COVID-19 risk: A population-based, cohort study. J. Endocrinol. Investig..

[27] Parant F., Bouloy J., Haesebaert J., Bendim’red L., Goldet K., Vanhems P., Henaff L., Gilbert T., Cuerq C., Blond E. (2022). Vitamin D and COVID-19 Severity in Hospitalized Older Patients: Potential Benefit of Prehospital Vitamin D Supplementation. Nutrients.

[28] Villasis-Keever M.A., López-Alarcón M.G., Miranda-Novales G., Zurita-Cruz J.N., Barrada-Vázquez A.S., González-Ibarra J., Martínez-Reyes M., Grajales-Muñiz C., Santacruz-Tinoco C.E., Martínez-Miguel B. (2022). Efficacy and Safety of Vitamin D Supplementation to Prevent COVID-19 in Frontline Healthcare Workers. A Randomized Clinical Trial. Arch. Med. Res..

[29] Romero-Ibarguengoitia M.E., Gutiérrez-González D., Cantú-López C., Sanz-Sánchez M., González-Cantú A. (2023). Effect of Vitamin D_3_ Supplementation vs. Dietary–Hygienic Measures on SARS-CoV-2 Infection Rates in Hospital Workers with 25-Hydroxyvitamin D_3_ [25(OH)D_3_] Levels ≥20 ng/mL. Microorganisms.

[30] Cangiano B., Fatti L.M., Danesi L., Gazzano G., Croci M., Vitale G., Gilardini L., Bonadonna S., Chiodini I., Caparello C.F. (2020). Mortality in an Italian nursing home during COVID-19 pandemic: Correlation with gender, age, ADL, vitamin D supplementation, and limitations of the diagnostic tests. Aging.

[31] Arroyo-Díaz J.A., Julve J., Vlacho B., Corcoy R., Ponte P., Román E., Navas-Méndez E., Llauradó G., Franch-Nadal J., Domingo P. (2021). Previous Vitamin D Supplementation and Morbidity and Mortality Outcomes in People Hospitalised for COVID-19: A Cross-Sectional Study. Front. Public Health.

[32] Cereda E., Bogliolo L., Lobascio F., Barichella M., Zecchinelli A.L., Pezzoli G., Caccialanza R. (2021). Vitamin D supplementation and outcomes in coronavirus disease 2019 (COVID-19) patients from the outbreak area of Lombardy, Italy. Nutrition.

[33] Ma H., Zhou T., Heianza Y., Qi L. (2021). Habitual use of vitamin D supplements and risk of coronavirus disease 2019 (COVID-19) infection: A prospective study in UK Biobank. Am. J. Clin. Nutr..

[34] Oristrell J., Oliva J.C., Subirana I., Casado E., Domínguez D., Toloba A., Aguilera P., Esplugues J., Fafián P., Grau M. (2021). Association of Calcitriol Supplementation with Reduced COVID-19 Mortality in Patients with Chronic Kidney Disease: A Population-Based Study. Biomedicines.

[35] Karonova T.L., Golovatyuk K.A., Kudryavtsev I.V., Chernikova A.T., Mikhaylova A.A., Aquino A.D., Lagutina D.I., Zaikova E.K., Kalinina O.V., Golovkin A.S. (2022). Effect of Cholecalciferol Supplementation on the Clinical Features and Inflammatory Markers in Hospitalized COVID-19 Patients: A Randomized, Open-Label, Single-Center Study. Nutrients.

[36] van Helmond N., Brobyn T.L., LaRiccia P.J., Cafaro T., Hunter K., Roy S., Bandomer B., Ng K.Q., Goldstein H., Mitrev L.V. (2022). Vitamin D_3_ Supplementation at 5000 IU Daily for the Prevention of Influenza-like Illness in Healthcare Workers: A Pragmatic Randomized Clinical Trial. Nutrients.

[37] Kaufman H.W., Niles J.K., Kroll M.H., Bi C., Holick M.F. (2020). SARS-CoV-2 positivity rates associated with circulating 25-hydroxyvitamin D levels. PLoS ONE.

[38] Grant W.B., Lahore H., McDonnell S.L., Baggerly C.A., French C.B., Aliano J.L., Bhattoa H.P. (2020). Evidence that Vitamin D Supplementation Could Reduce Risk of Influenza and COVID-19 Infections and Deaths. Nutrients.

[39] Sabetta J.R., DePetrillo P., Cipriani R.J., Smardin J., Burns L.A., Landry M.L. (2010). Serum 25-Hydroxyvitamin D and the Incidence of Acute Viral Respiratory Tract Infections in Healthy Adults. PLoS ONE.

[40] Charoenngam N., Shirvani A., Reddy N., Vodopivec D.M., Apovian C.M., Holick M.F. (2021). Association of Vitamin D Status with Hospital Morbidity and Mortality in Adult Hospitalized Patients with COVID-19. Endocr. Pract..

[41] Liu Y., Clare S., D’erasmo G., Heilbronner A., Dash A., Krez A., Zaworski C., Haseltine K., Serota A., Miller A. (2023). Vitamin D and SARS-CoV-2 Infection: SERVE Study (SARS-CoV-2 Exposure and the Role of Vitamin D among Hospital Employees). J. Nutr..

[42] Martineau A.R. (2023). Vitamin D in the prevention or treatment of COVID-19. Proc. Nutr. Soc..

[43] Shah K., Varna V.P., Sharma U., Mavalankar D. (2022). Does vitamin D supplementation reduce COVID-19 severity? A systematic review. QJM Int. J. Med..

[44] Hariyanto T.I., Intan D., Hananto J.E., Harapan H., Kurniawan A. (2022). Vitamin D supplementation and COVID-19 outcomes: A systematic review, meta-analysis and meta-regression. Rev. Med. Virol..

[45] Ødum S.F., Kongsbak-Wismann M. (2023). Vitamin D and SARS-CoV-2. Basic Clin. Pharmacol. Toxicol..

[46] Hu X. (2023). The Effect of Active Vitamin D on Coronavirus. Highlights Sci. Eng. Technol..

[47] Pal R., Banerjee M., Bhadada S.K., Shetty A.J., Singh B., Vyas A. (2022). Vitamin D supplementation and clinical outcomes in COVID-19: A systematic review and meta-analysis. J. Endocrinol. Investig..

[48] Beran A., Mhanna M., Srour O., Ayesh H., Stewart J.M., Hjouj M., Khokher W., Mhanna A.S., Ghazaleh D., Khader Y. (2022). Clinical significance of micronutrient supplements in patients with coronavirus disease 2019: A comprehensive systematic review and meta-analysis. Clin. Nutr. ESPEN.

[49] Wang J., Feng M., Ying S., Zhou J., Li X. (2018). Efficacy and Safety of Vitamin D Supplementation for Pulmonary Tu-berculosis: A Systematic Review and Meta-analysis. Iran. J. Public Health.

[50] Charan J., Goyal J.P., Saxena D., Yadav P. (2012). Vitamin D for prevention of respiratory tract infections: A systematic review and meta-analysis. J. Pharmacol. Pharmacother..

[51] Autier P., Gandini S. (2007). Vitamin D Supplementation and Total Mortality: A Meta-Analysis of Randomized Controlled Trials. Arch. Intern. Med..

[52] Parisini A., Boni S., Vacca E.B., Bobbio N., Del Puente F., Feasi M., Prinapori R., Lattuada M., Sartini M., Cristina M.L. (2023). Impact of the COVID-19 Pandemic on Epidemiology of Antibiotic Resistance in an Intensive Care Unit (ICU): The Experience of a North-West Italian Center. Antibiotics.

[53] Petrakis V., Panopoulou M., Rafailidis P., Lemonakis N., Lazaridis G., Terzi I., Papazoglou D., Panagopoulos P. (2023). The Impact of the COVID-19 Pandemic on Antimicrobial Resistance and Management of Bloodstream Infections. Pathogens.

